# Efficient adsorptive removal of Congo Red dye using activated carbon derived from *Spathodea campanulata* flowers

**DOI:** 10.1038/s41598-025-86032-9

**Published:** 2025-01-13

**Authors:** Sujesh Sudarsan, Gokulakrishnan Murugesan, Thivaharan Varadavenkatesan, Ramesh Vinayagam, Raja Selvaraj

**Affiliations:** 1 Department of Chemical Engineering, Manipal Institute of Technology, Manipal Academy of Higher Education, Manipal, Karnataka 576104 India; 2 Department of Biotechnology, M.S.Ramaiah Institute of Technology, Bengaluru, Karnataka 560054 India; 3 Department of Biotechnology, Manipal Institute of Technology, Manipal Academy of Higher Education, Manipal, Karnataka 576104 India

**Keywords:** Adsorption, Activated carbon, *Spathodea campanulata*, Congo red dye, Regeneration, Spiking studies, Chemistry, Chemical engineering, Environmental chemistry, Materials chemistry

## Abstract

This report investigates the preparation, characterization, and application of activated carbon derived from *Spathodea campanulata* flowers (SCAC) to remove Congo Red (CR) dye from aqueous streams. SCAC was synthesized using orthophosphoric acid activation which yielded a mesoporous material with a specific surface area of (986.41 m^2^/g), significantly exceeding values reported for flower-derived activated carbons in the available literature. Field emission scanning electron microscopy (FESEM) image revealed an irregular, rough surface morphology pre-adsorption, which became smoother post-adsorption, indicating successful CR attachment. Elemental analysis through energy-dispersive x-ray spectroscopy (EDS) and X-ray photoelectron spectroscopy (XPS) confirmed an increase in carbon content and the appearance of sulfur, verifying CR uptake. Adsorption kinetics obeyed the pseudo-second-order equation, signifying chemisorption, while the equilibrium dataset fitted better to the Langmuir model, with R^2^ of 0.9944, suggesting a monolayer adsorption mechanism with a maximum adsorption capacity of 59.27 mg/g. Thermodynamic analysis revealed spontaneous and endothermic adsorption process. Desorption studies showed methanol as the most effective desorbing agent, with SCAC retaining considerable adsorption capacity across six cycles, highlighting its reusability. In tests with real water matrices, SCAC demonstrated significantly higher removal efficiency in natural waters than control, suggesting enhanced adsorption in complex matrices. These findings underscore the practical applicability of SCAC in real-world wastewater treatment, offering a promising solution for large-scale industrial applications.

## Introduction

Environmental pollution, encompassing air, soil, and water, represents a significant challenge in the modern era^1^. Rapid industrialization, urbanization, and population growth have led to the increased release of pollutants into the environment, impacting human health, wildlife, and natural ecosystems^2^. Among these, water pollution from industries stands out as a critical issue, which contaminates water bodies, rendering them unsafe for consumption and harming aquatic life^3^. Industrial wastewater often contains a complex mixture of pollutants, including heavy metals, pharmaceuticals, pesticides, and various dyes^4^.

To mitigate the detrimental impacts of these contaminants, different treatment methods have been explored, encompassing physical, chemical, and biological approaches. While physical treatments like sedimentation and filtration offer cost-effective removal of particulate pollutants, their efficacy for dissolved contaminants is limited, often necessitating further treatment steps^5^. Conversely, chemical methods, including coagulation, flocculation, and oxidation, demonstrate higher efficiency in pollutant removal^6^. However, these methods are expensive, energy-intensive, and may generate sludge. Biological methods often encounter challenges such as slow degradation rates, limited effectiveness against diverse pollutant structures, and sensitivity to environmental parameters, hindering their scalability for industrial applications^7^. The difficulty in treating industrial effluent is exacerbated by the presence of synthetic dyes, which are extensively used in the textile industry due to their vibrant colors and chemical stability.

The textile industry, a major global water consumer, relies on various dye types, including reactive, direct, acid, and dispersed dyes^8^. Congo Red (CR), a synthetic diazo dye characterized by its vibrant red color, has a long history in textile industry. CR dye possesses sulfonic acid groups (–SO_3_^–^) that ionize in solution, giving it a net negative charge^9^. The discharge of CR into water bodies poses severe environmental and health risks. These dyes disrupt aquatic ecosystems by altering water properties, promoting eutrophication, and inhibiting plant photosynthesis, ultimately harming aquatic life^10^. Additionally, CR raises significant health concerns including the development of cancerous cells, especially in the bladder^11^. As a result, the development of efficient and sustainable treatment methods for the removal of CR and similar dyes from wastewater has become a critical area of research.

Recently, adsorption, a surface phenomenon, has garnered significant attention for its effectiveness in removing a wide range of pollutants, including CR from wastewater. This method is a preferred choice due to its simple process, inexpensive nature, minimum sludge production, selective removal capabilities, and high efficiency^12^. Various adsorbents, such as clay minerals, agricultural by-products, zeolites, biochar, and MOFs, have been studied for CR removal, but their practical use is often limited by high costs and low stability in water^13^.

Activated carbon (AC) has garnered substantial attention in the adsorption process due to its numerous distinct advantages. Its high surface area, resulting from its porous structure, provides abundant binding sites for adsorbate molecules such as CR, making it highly efficient for a wide range of environmental and industrial applications. Furthermore, the inherent regenerative capacity of AC, achievable through various treatment methods, significantly enhances its economic and environmental sustainability. Additionally, these materials are considered environmentally benign, with minimal ecological impact and potential for sustainable production from various carbonaceous sources, including coconut shells^14^, rice husk^15^, and corn cobs^16^ among the recent examples.

Despite its advantages, AC derived from these precursors presents significant limitations. In particular, AC synthesized from coconut shells achieved a high surface area of 1550 m^2^/g through pyrolysis at 900 ℃ under a nitrogen atmosphere^14^. However, its maximum adsorption capacity for CR remained comparatively low at 22.1 mg/g. Furthermore, the high energy demands associated with such elevated pyrolysis temperatures pose challenges to the cost-effectiveness of large-scale AC production, limiting its practical feasibility. Similarly, while rice husk offers a cost-effective and abundant feedstock, the resulting AC demonstrated a considerably lower adsorption capacity of 11.84 mg/g, hindering its real-world applicability^15^. Besides, AC produced from corn cobs achieved a moderate surface area of 650 m^2^/g, yet its maximum adsorption capacity was only 17.89 mg/g^16^. These findings highlight the persistent challenge of achieving a balance between high surface area and superior adsorption performance in AC produced from conventional biomass sources.

Given these limitations, exploring alternative biomass sources with unique properties, such as *Spathodea campanulata* flowers, could offer a promising avenue for preparing AC with improved CR adsorption capabilities. *S. campanulata*, well-known as the African tulip tree, is an ornamental tree and widely cultivated in subtropical regions, including India. Recent studies have explored the use of *S. campanulata* biomass for AC preparation to remove various dyes from aqueous solutions. For instance, AC prepared using the *S. campanulata* leaves exhibited a low adsorption potential of 11.73 mg/g for CR dye^17^. Another study explored the use of AC derived from *S. campanulata* stems to remove methylene blue^18^. Although various parts of *S. campanulata* have been extensively studied for AC synthesis, its flowers remain an untapped resource, offering a significant opportunity that this study seeks to explore. The abundance and rapid growth of *S. campanulata* in subtropical regions make it a readily available and sustainable biomass source. Utilizing its flowers, often treated as waste, contributes to waste valorisation and fosters a circular economy by reducing environmental burdens while promoting eco-friendly processes. These flowers are uniquely rich in phytochemicals such as flavonoids, tannins, lignin, and other bioactive compounds, creating abundant active sites for efficient CR adsorption^19^.

The study also addresses the limitations of existing ACs derived from various biomass sources, which often face challenges such as high production costs, complex synthesis procedures, low surface area and inadequate adsorption capacity. By utilizing *S. campanulata* flowers as a precursor and orthophosphoric acid (H_3_PO_4_) as the activating agent, our method effectively overcomes these limitations. Specifically, the use of *S. campanulata* flowers, reduces production costs by providing an inexpensive and abundant raw material. Additionally, H_3_PO_4_ simplifies the synthesis process as it is less corrosive and safer to handle^20^ compared to alternatives like H_2_SO_4_, ZnCl_2_, or KOH. These alternatives necessitate higher activation temperatures in the range of 600‒ 900℃ and inert atmospheres, increasing energy consumption and safety risks^21,22^. In contrast, our method, employing a 1:1 biomass‒to‒H_3_PO_4_ ratio, operates efficiently at a lower temperature (400 ℃ for 2 h) without the need for an inert atmosphere. This approach reduces energy costs and enhances safety while achieving a high surface area and improved adsorption capacity. Furthermore, the use of H_3_PO_4_ contributes to the sustainability of the process due to its lower toxicity and reduced environmental hazards^23^. Also, a subsequent neutralization step with an appropriate base ensures the safe handling and disposal of the spent activating agent, further minimizing environmental impact.

Hence, the present investigation focuses on the preparation and application of AC derived from the sustainable and readily available biomass source, *S. campanulata* flowers, for the removal of CR dye. Kinetics and isotherm studies were employed to gain mechanistic insights into the adsorption process, elucidating the interactions between CR dye molecules and the AC surface. In addition, the study thoroughly examines the desorption and regeneration potential of SCAC, demonstrating its ability to maintain efficiency over multiple reuse cycles and highlighting its economic and environmental viability. To evaluate its practical applicability, SCAC was also tested in real-world water matrices, confirming its effectiveness in treating contaminated water under realistic conditions. These findings establish *S. campanulata* flowers as a valuable precursor for AC synthesis, offering efficient and sustainable solutions for wastewater treatment challenges.

## Materials and methodology

### Materials

*Spathodea campanulata* flowers were gathered within the university campus in Manipal, India. CR dye (C_32_H_22_N_6_Na_2_O_6_S_2_, MW: 696.66 g/mol) was procured from Himedia (India). Orthophosphoric acid (H_3_PO_4,_ 85%) and sodium bicarbonate (NaHCO_3_) were obtained from Sigma-Aldrich (USA) and Merck (Germany) respectively. HCl and NaOH were acquired from Fisher Scientific (USA). All experiments were conducted using distilled water.

### Adsorbent preparation

**Fig. 1:**
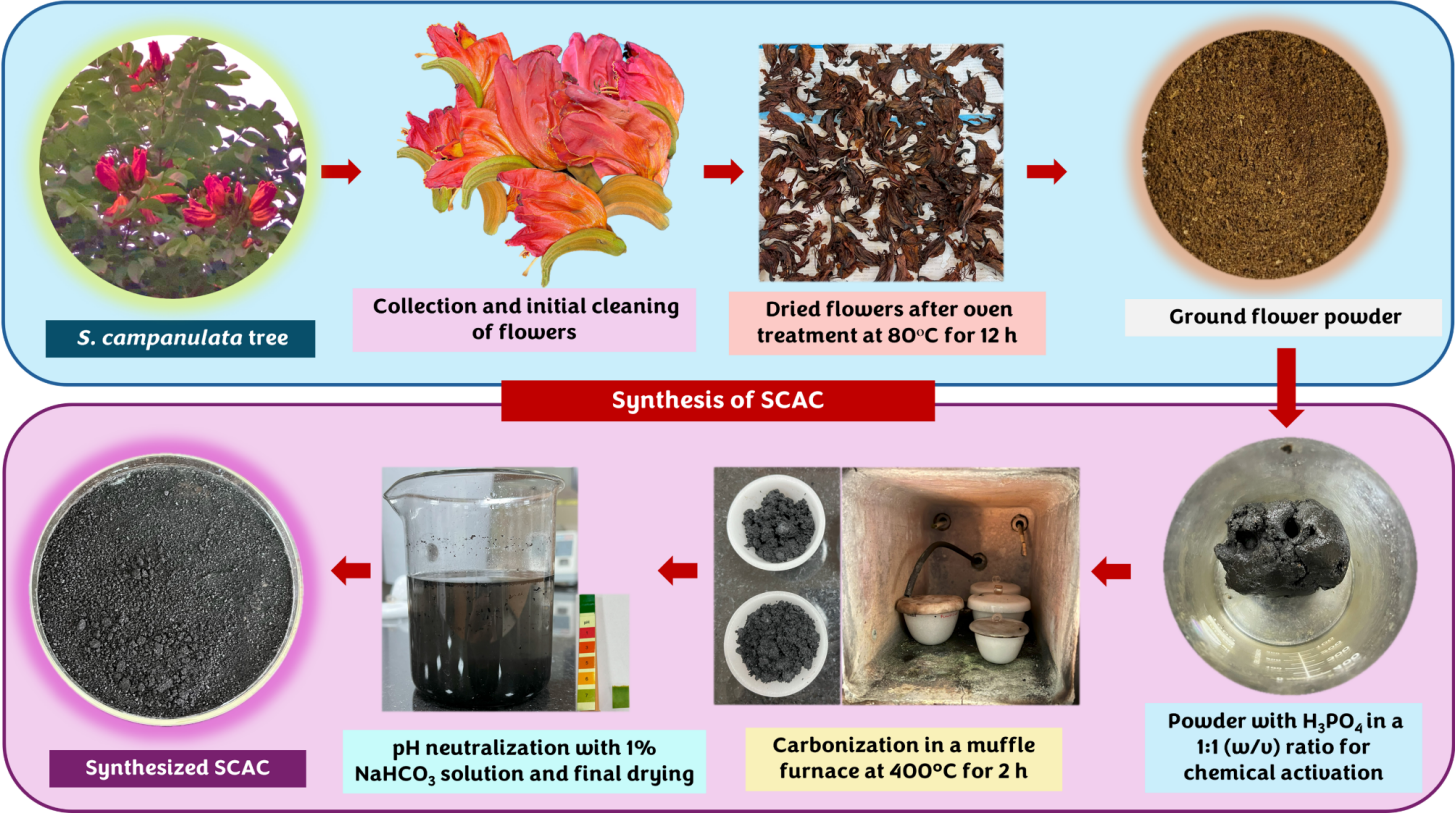
Schematic representation of the synthesis process for SCAC derived from *S. campanulata* flowers.

AC was synthesized from *S. campanulata* flowers, following a modified method previously described by Patra et al.^24^. The flowers of the tree were taxonomically identified and authenticated by Ms. Gayathri Pai, Department of Botany, MGM College, Udupi, India. In brief, *S. campanulata* flowers were collected, cleaned with distilled water, and then dried (hot air oven: 80 ℃, 12 h). Subsequently, the dried flowers were powdered. For chemical activation, the powder was blended with H_3_PO_4_ at 1:1 (w/v) and left undisturbed for 6 h. Subsequently, the contents were placed for ageing (hot air oven: 80 ℃, 12 h). The contents were combusted in a muffle furnace (400 ℃, 2 h). The carbonized material was washed thoroughly with 1% (w/v) NaHCO_3_ solution till neutral pH, dried (hot air oven: 100 ℃, 12 h), and the prepared AC, was labeled as SCAC (Fig. 1).

### Structural and functional analysis of SCAC

Several techniques were employed to characterize the synthesized SCAC. The surface morphological features and elemental compositions were analyzed by Field Emission Scanning Electron Microscopy (FESEM, Zeiss Sigma 300, Carl Zeiss, Germany) integrated with Energy-Dispersive X-ray Spectroscopy (EDS, Oxford Instruments, UK). The specific surface area (SSA) and pore volume were measured using Brunauer-Emmett-Teller (BET, Smart instrument, India) analysis. Additionally, the crystalline nature was investigated using X-ray Diffraction (XRD, D8 Advance, Bruker, Germany). The surface nature and bonding types were examined using X-ray Photoelectron Spectroscopy (XPS, ThermoFisher, UK). Finally, the functional moieties were investigated by Fourier Transform Infrared Spectroscopy (FTIR, Shimadzu 8400 S, Japan). The point–of–zero charge (pH_PZC_) was estimated by pH drift methodology.

### Adsorption experiments

Adsorption experimentations were performed to assess the efficacy of SCAC to remove CR. To begin, the pH influence was investigated by introducing 0.5 g/L SCAC into 100 mL of 50 mg/L CR dye, with pH values between 5 and 10 (CR dye changes color and structure due to protonation at pH < 5). Following the pH study, the influence of SCAC dosage on CR adsorption was analyzed. Varying SCAC dosages between 0.2 and 1.0 g/L, were mixed with 50 mg/L CR dye while maintaining the optimized pH. Subsequently, the influence of initial CR concentrations and contact duration were investigated at CR concentrations between 20 and 60 mg/L, using the optimal pH and dosage. In addition to these parameters, the effect of temperature was also evaluated. The temperature was changed between 293 and 323 K under the optimal pH, dosage, and CR concentration to assess its effect on CR removal. Throughout all experiments, the mixtures were constantly shaken (150 rpm, 30 ℃) in a temperature-controlled shaker (Remi CIS-24 Plus). At predetermined intervals, samples were collected and centrifuged (Eppendorf Centrifuge 5425, Germany). The residual CR concentration was measured by UV-visible spectrometer (Shimadzu UV-1900i) at 497 nm. Triplicate experiments were done, and mean values were reported. The removal efficiency (R, %) and adsorption capacity (q_e,_ mg/g) were calculated by Eqs. (1) and (2).

1 $$R = \frac{{C_{i} - C_{t} }}{{C_{i} }} \times 100$$

2 $$q_{e} = \frac{{\left( {C_{i} - C_{e} } \right)V}}{m}$$

​ 

wherein *C*_*i*_, *C*_*t*_, and *C*_*e*_ are the CR concentrations at initial, time ‘*t’*, and equilibrium (mg/L). *V* represents the volume of the solution (L), and *m* denotes the mass of SCAC (g).

### Adsorption modeling

#### Adsorption kinetics

The adsorption kinetics were performed to examine the rate-controlling step. The kinetic dataset was analyzed using well-known models: pseudo-first-order (PFO), pseudo-second-order (PSO), and intraparticle diffusion (IPD) model^25^, as represented by Eqs. (3)-(5).

3 $${\text{PFO}}:q_{t} = q_{e} (1 - exp^{{ - k_{1} t}} )$$

4 $${\text{PSO}}:q_{t} = \frac{{q_{e}^{2} k_{2} t}}{{1 + q_{e} k_{2} t}}$$

5 $$q_{t} = K_{{diff}} t^{{0.5}} + C$$

wherein, *q*_*t*_ and *q*_*e*_ represent adsorption capacity at time ‘t’ and equilibrium correspondingly. *k*_*1*_, *k*_*2*_, and *K*_*diff*_ designate respective PFO, PSO and IPD rate constant values. C is the IPD intercept.

#### Adsorption Isotherm

To analyze the adsorption mechanism and evaluate the adsorption efficacy of SCAC, the experimental dataset was fitted to isotherm models: Langmuir, Freundlich, and Temkin^26^ as given by Eqs. (6)-(8).

6 $${\text{Langmuir: }}\:q_{e} = \frac{{q_{m} \:K_{L} \:C_{e} }}{{(1 + \:K_{L} \:C_{e} )}}$$

7 $${\text{Freundlich: }}\:q_{e} = K_{F} C_{e}^{{1/n}}$$

8 $${\text{Temkin: }}\:q_{e} = B_{T} ln\left( {K_{t} C_{e} } \right)$$

where, *q*_*m*_: maximum adsorption capacity (mg/g), *K*_*L*_: Langmuir constant (L/mg), *K*_*F*_: Freundlich constant ((mg/g)/(L/mg)^1/n^), *1/n*: Freundlich exponent, *B*_*T*_: heat of adsorption constant (J/mol) and *K*_*t*_: equilibrium binding constant (L/mg).

#### Adsorption thermodynamics

To examine the feasibility, spontaneity, and adsorption nature, the thermodynamic factors like change in standard enthalpy (ΔH°), standard entropy (ΔS°), and standard Gibbs free energy (ΔG°) were calculated by using the Van’t Hoff model^27^ as given by Eq. (9).

9 $${\text{Van}}\text{'} {\text{t Hoff equation}}:K_{T} = exp\left[ {\left( {\frac{{\Delta S^{{}} }}{R}} \right) - \left( {\frac{{\Delta H^{{}} }}{R}} \right)\frac{1}{T}} \right]$$

Where, *K*_*T*_ = *q*_*e*_*/C*_*e*_: Equilibrium constant (L/g), *R*: Ideal gas constant and T: Absolute temperature (K) and *ΔG*^*ο*^*= – RT ln K*_*T*_.

### Desorption and regeneration potential of SCAC

Desorption experimentations were done to examine the regeneration potential of spent SCAC, aiming to enhance the economic feasibility. Four desorbing agents such as methanol, ethanol, NaOH (0.1 N), and HCl (0.1 N) were assessed for their efficiency in desorbing CR. Spent SCAC, obtained from adsorption experiments with 20 mg/L CR dye and 0.5 g/L SCAC, was treated with 50 mL of each desorbing agent. After desorption, the SCAC was harvested, dried (80 ℃, 2 h) and stored for reuse. To assess the effectiveness of each desorbing agent, a second adsorption cycle was performed with the regenerated SCAC. The desorbing agent demonstrating the highest CR removal was selected for further reusability studies, wherein six consecutive adsorption-desorption series were performed. Following every cycle, the adsorption performance of the regenerated SCAC was determined.

### Evaluating the efficacy of SCAC for removing CR from water matrices

Water samples from five distinct matrices namely, Arabi Falls, Tap water, Manipal Lake, and Suvarna River were collected via grab sampling and subsequently spiked with CR dye to assess the adsorbent’s efficiency under real-world conditions^28^. Industrial groundwater, sourced from a well within a textile industrial zone, was also considered in this study. This groundwater is likely to contain a variety of dissolved ions and other substances characteristic of industrial wastewater discharge or runoff, making it distinct from other natural water sources like rivers or lakes. Its inclusion in the study provides a realistic test case for SCAC in a complex, potentially contaminated, water matrix relevant to industrial settings. For each matrix, batch experiments were performed in triplicate to ensure accuracy and reproducibility. A fixed SCAC dosage of 0.5 g/L was added to 100 mL of each sample, spiked with 50 mg/L CR. The pH was standardized to 7.0 across all samples. The performance of SCAC was evaluated as discussed in the previous sections.

## Results and discussions

### Characterization studies of SCAC

#### Morphology and elemental features of SCAC

**Fig. 2:**
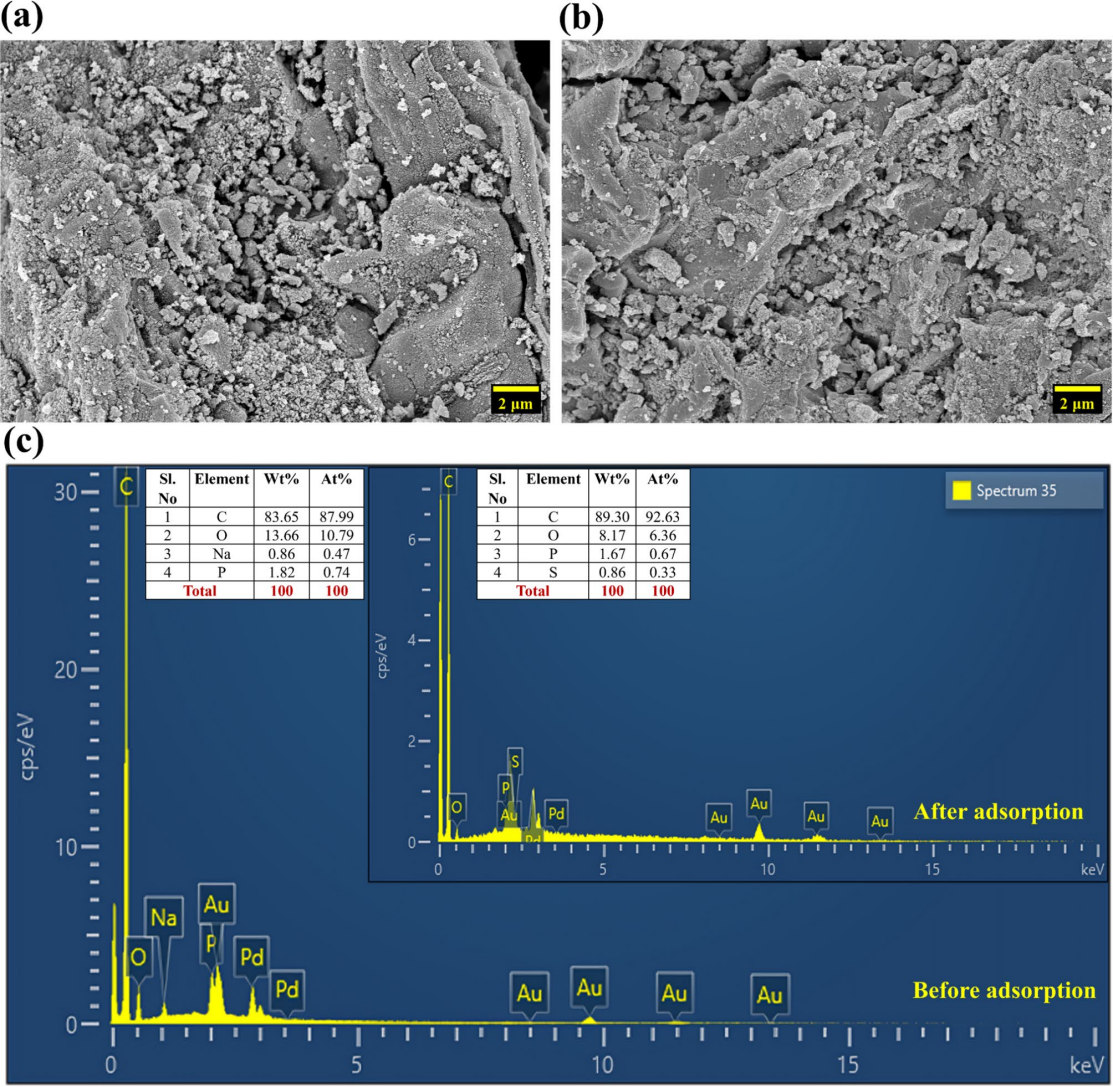
FESEM image of SCAC (**a**) before and (**b**) after adsorption; (**c**) EDS spectra of SCAC before and after adsorption.

FESEM images provided a detailed visual analysis of the morphological changes in SCAC before and after adsorption. The pre-adsorption image (Fig. 2a) revealed a highly irregular and rough surface morphology, characterized by a porous and flaky structure typical of lignocellulosic materials. Large, uneven cracks, voids, and granular debris were prevalent, all contributing to the high surface roughness and enhancing the available surface area for adsorption as documented in the literature^29^. Furthermore, the presence of well-developed pores confirmed the effectiveness of orthophosphoric acid activation in creating a hierarchical porous network, ideal for dye diffusion^30^. The SCAC surface underwent significant morphological transformations upon adsorption (Fig. 2b). The initially rough and irregular texture became smoother, indicating the successful adhesion of CR molecules. This transformation was further supported by the reduced visibility of cracks, voids, and pore openings.

EDS was performed to analyze the elemental nature of SCAC (Fig. 2c). Before adsorption, the SCAC exhibited predominant peaks for C and O, which ascertained the formation of AC. Additionally, phosphorus introduced during orthophosphoric acid activation, was initially present in trace amounts. The emergence of sulfur at 0.33% after adsorption provides direct evidence of successful CR uptake as reported in the literature^31^, since sulfur atoms are present in the molecular structure of CR.

#### Brunauer-Emmett-Teller analysis

The SSA and pore volume of SCAC in this study were measured as 986.41 m²/g and 0.8797 cc/g, respectively. This value stands out as high, especially when compared to recent literature reports on ACs derived from various floral sources. For instance, *Borassus aethiopum* flowers achieve a modest surface area of 9.57 m²/g^32^, *Hibiscus rosa-sinensis* yields 12.11 m²/g^33^, while marigold flowers attain a higher but still much lower value of 183 m²/g^34^. Even the male flowers of the coconut tree, which exhibit one of the more substantial surface areas among similar studies, reach only 328.2 m²/g^35^. The high surface area observed in SCAC is attributed to the chemical activation process using H_3_PO_4_, which promotes the development of a porous structure by breaking down biomass components and facilitating pore formation^36^. The contrast emphasizes SCAC’s potential in applications requiring high surface area and porosity, placing it well above the conventional floral-derived ACs in terms of performance. Additionally, the pore diameter of SCAC measured at 3.56 nm reveals the mesoporous nature of the adsorbent, which enhances its ability to adsorb CR molecules by providing greater pore accessibility.

#### XRD analysis

**Fig. 3:**
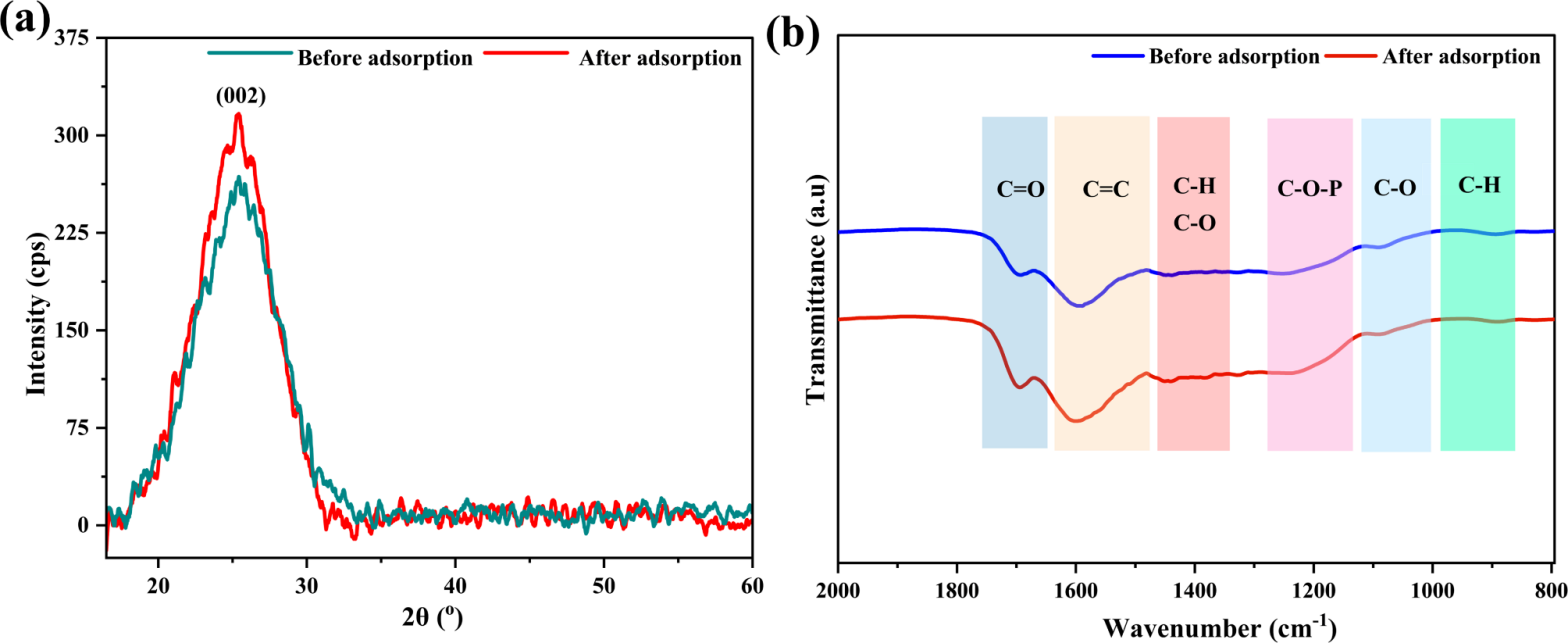
(**a**) XRD patterns of SCAC before and after adsorption; (**b**) FTIR spectra of SCAC before and after adsorption.

The broad XRD peak (Fig. 3a) observed between 2θ values of 10–40^ο^ corresponds to the amorphous phase commonly found in AC^37^. The XRD analysis revealed one prominent peak at a 2θ value of 25.46^ο^corresponding to the (002) plane, which indicates the graphite crystallites^38^ within the AC structure. The XRD peaks observed in this study align with findings by Prakash et al.., who identified a peak at a 2θ value of 26^ο^, confirming the presence of graphite in porous AC derived from arhar stalks^39^. Similarly, Somsesta et al. reported an XRD peak at a 2θ value of 25^ο^ for AC prepared from sisal fiber^40^.

Interestingly, the diffractograms for the (002) plane showed no significant changes in peak position before and after adsorption, suggesting that the overall crystallinity of the SCAC structure remained unaffected throughout the adsorption process. However, a slight increase in the sharpness and intensity of the peaks was observed after adsorption, suggesting that the adsorption of CR may induce subtle rearrangement or modifications on the SCAC, leading to a more ordered structure and consequently sharper peaks. Another possible reason for the increase in sharpness and intensity is the intercalation of CR molecules between the graphitic layers of SCAC^41^.

#### FTIR analysis

FTIR spectra revealed key changes before and after adsorption, providing insights into the adsorption mechanism (Fig. 3b). Before adsorption, the FTIR spectrum exhibited a peak at 1691.57 cm^− 1^ suggesting carbonyl groups, possibly from aldehydes, ketones, or carboxylic acids^42^. Additionally, the peak at 1589.34 cm^− 1^ is indicative of C = C stretching vibration, typically associated with aromatic rings, supporting the presence of aromatic structures within SCAC. The 1448.54 cm^− 1^ signal relates to both C-H bending vibration and C-O stretching vibrations^43^. Furthermore, the signal at 1253.73 cm^− 1^ is credited to C–O stretching from alcohols, ethers, and C–O–P stretching introduced by orthophosphoric acid, confirming the activation of SCAC with acid-derived functional moieties. The band at 1091.71 cm^− 1^, characteristic of C–O stretching suggests the presence of alcohols, ethers, or esters^44^. Finally, the peak at 896.90 cm^− 1^ is because of the out–of–plane C–H bending, commonly associated with aromatic rings or alkenes^43^.

After CR adsorption, several key changes occurred in the FTIR spectrum (Fig. 3b**)**. For instance, the slight shifts in the C = O stretching peak at 1693.50 cm^− 1^ points to interactions between CR and the carbonyl groups on the carbon surface^42^. The shift of the C = C stretching signal at 1600.92 cm^− 1^ suggests interactions between CR and the aromatic rings of SCAC, possibly through π–π interactions. Furthermore, a subtle shift in the C–H bending peak at 1450.47 cm^− 1^ may result from the influence of the adsorbed CR on the SCAC surface. Also, the shift in the peak at 1244.09 cm^− 1^, originally at 1253.73 cm^− 1^, suggests interactions between CR and the functional groups responsible for this vibration in SCAC, possibly involving C–O or C–H bonds^45^. The consistent presence of a 1091.71 cm^− 1^ signal indicates that the C–O stretching vibration is not significantly affected by the adsorption process. In addition to these shifts, it is also important to note that the peak intensity reduced post-adsorption. This reduction is primarily due to surface coverage by CR molecules, which masks the vibrational modes of surface groups present in SCAC. Moreover, interactions like hydrogen bonds and π–π interaction among CR and the SCAC alter the electron distribution and restrict the vibrational freedom of the surface groups, further contributing to the decrease in peak intensity.

#### XPS studies

**Fig. 4:**
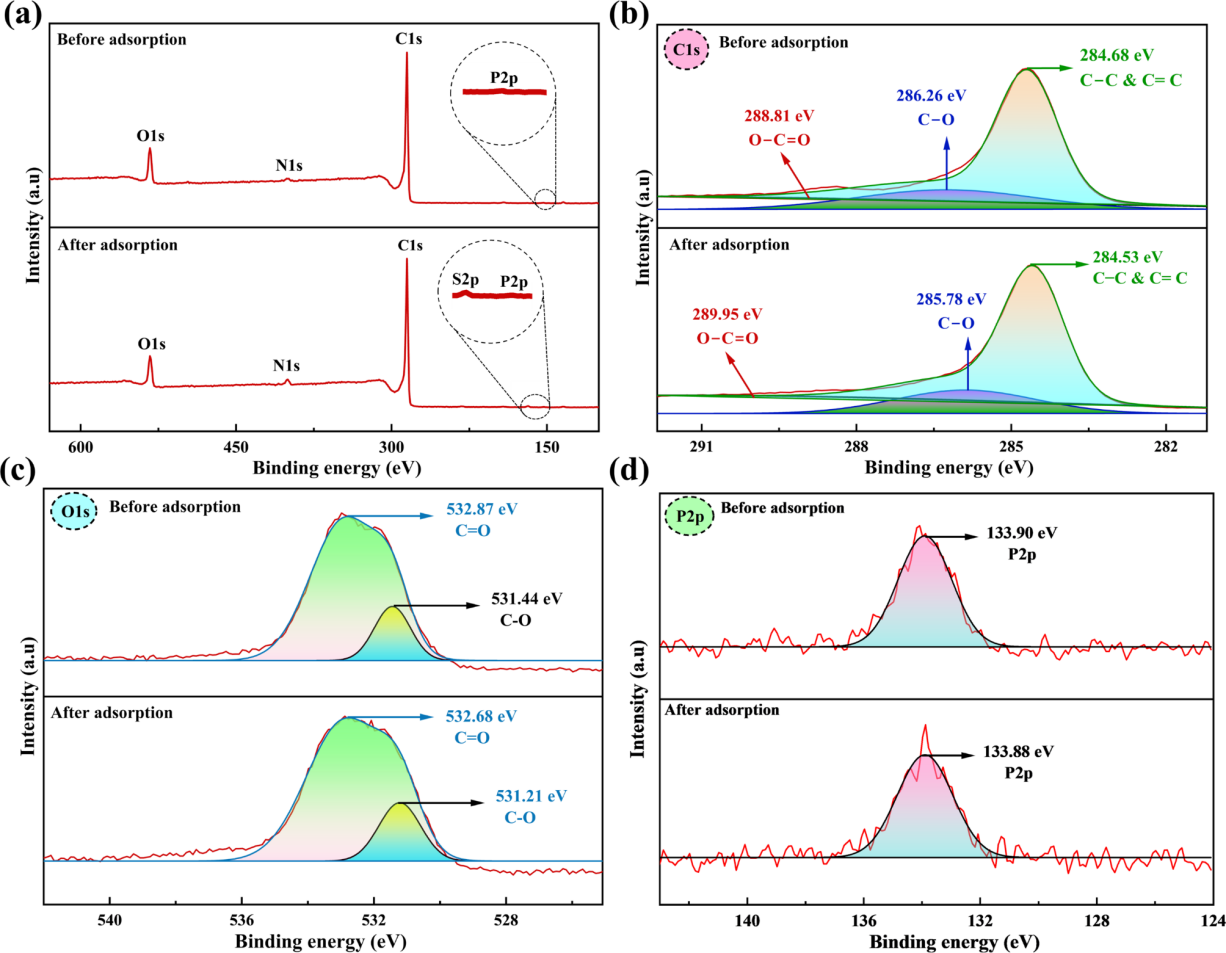
XPS spectra of SCAC before and after adsorption, showing detailed analysis of (**a**) full survey spectrum (**b**) C1s, (**c**) O1s and (**d**) P2p spectra.

XPS survey of SCAC shown in Fig. 4a revealed prominent peaks for carbon (C1s), oxygen (O1s), nitrogen (N1s), and phosphorus (P2p) (Fig. 4a inset), confirming their presence on the surface of SCAC. After adsorption, the peak corresponding to sulfur (S2p) (Fig. 4a inset), appeared, implying the successful binding of CR onto SCAC. The high intense sharp C1s peak indicates the high carbon content of SCAC, while the O1s peak represents the presence of oxygen-containing functional groups formed or modified during thermal activation. The P2p peak (Fig. 4d) signifies phosphatic phosphorus, likely incorporated from the orthophosphoric acid used in the synthesis process^46^.

Specifically, the high-resolution XPS spectrum of C1s before adsorption features a broad peak that has been deconvoluted into three distinct components (Fig. 4b). The signal at 284.68 eV relates to C–C/C = C bonds, representing pure or unfunctionalized carbon^47^. After the adsorption of CR, a minor shift of the C–C/C = C signal to 284.53 eV was observed, possibly due to the interactions with CR molecules^48^. Meanwhile, the 286.26 eV signal is ascribed to C–O bonds shifted to 285.78 eV pointing to the involvement of these groups in adsorption. Furthermore, the peak at 288.81 eV is associated with O–C = O bonds, representing the existence of carboxylic groups formed during AC preparation^49^. After the adsorption of CR, this peak shifted to a higher binding energy of 289.95 eV, suggesting the alterations in their chemical environment due to interactions with CR molecules.

In the O1s spectrum before CR adsorption (Fig. 4c), two deconvoluted peaks were observed: one at 532.87 eV, representing C = O linkages, and another at 531.44 eV, corresponding to C–O bonds^47^. After adsorption, both the C–O and C = O peaks moved slightly to low binding energy values of 532.68 and 531.21 eV attributing their involvement in the adsorption process, which aligns with the shifts observed in the C1s spectra for the C–O bonds. Additionally, the P2p spectrum before CR adsorption in Fig. 4d portrayed a signal at 133.9 eV, indicating the integration of phosphorus within the carbon matrix, primarily in the form of phosphates and polyphosphates^46^. The P2p peak remained relatively unchanged after adsorption, suggesting that phosphate groups might not be directly participating in the adsorption but contribute to the structural stability of the SCAC material. The binding energies of C1s, O1s, and P2p align with values reported in the literature for AC synthesized from *Peltophorum pterocarpum* pods^50^ and *Ficus religiosa* leaves^46^.

The elemental composition of SCAC before adsorption revealed 78.38% carbon, 9.61% oxygen, and 1.16% nitrogen. After adsorption, the carbon content slightly decreased to 77.66%, likely due to CR molecules masking the SCAC surface and attenuating the signal from underlying carbon atoms. Similarly, oxygen content also showed a minor reduction to 9.20%, possibly due to surface coverage by CR, interactions with oxygen-containing groups, or displacement of some oxygen species. The XPS and EDS findings thus reveal key changes in elemental composition, including variations in carbon and oxygen levels and the appearance of sulfur after adsorption. FTIR analysis further supports these results, showing shifts in functional moieties such as C = C, C–O, C = O, and C–H, that actively participate in the adsorption process. Together, these observations indicate enhanced CR uptake driven by mechanisms such as electrostatic interactions, hydrogen bonding, or a combination of both.

### Adsorption experimentation studies

#### pH effect

**Fig. 5:**
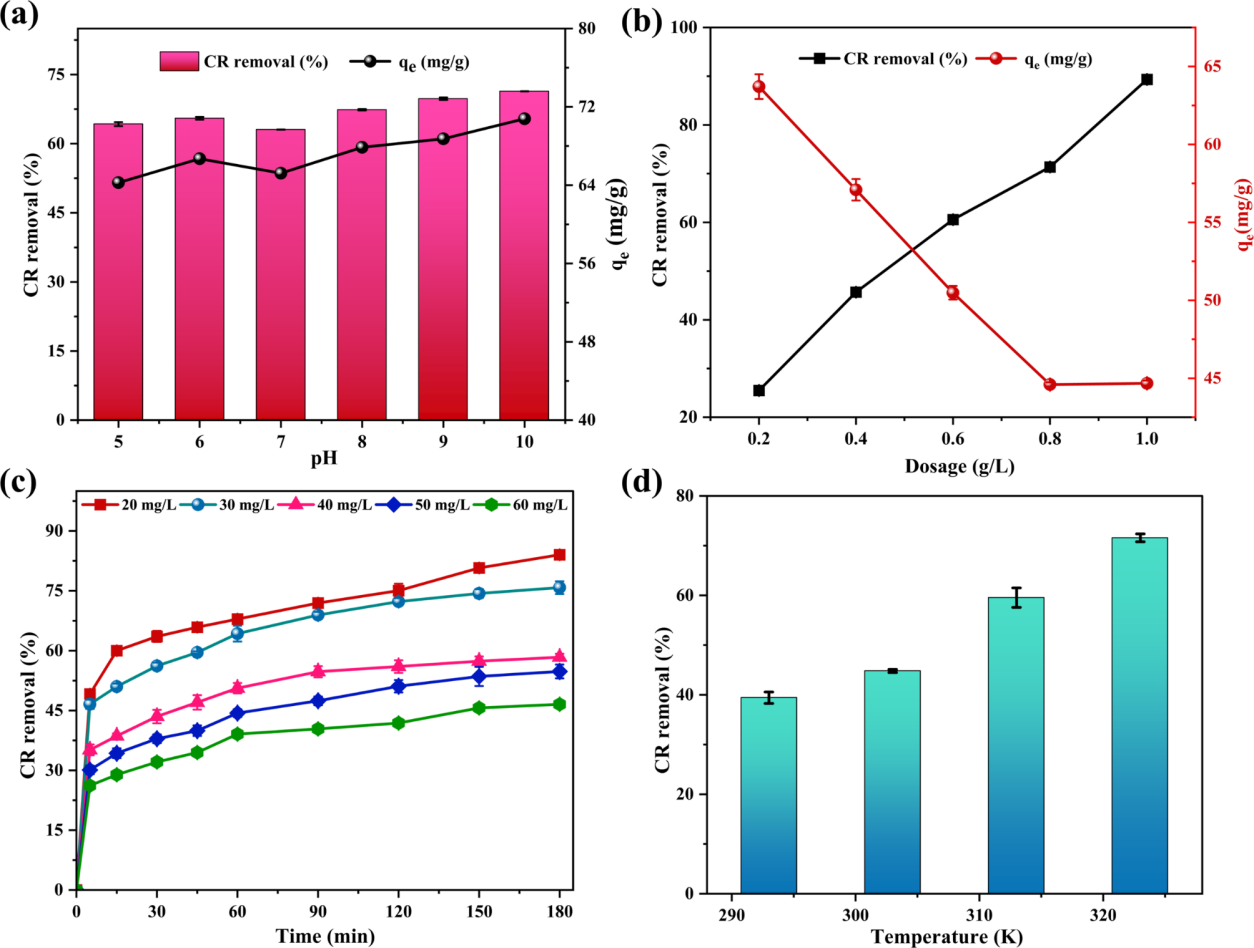
(**a**) Adsorption parameters including (**a**) influence of pH, (**b**) effect of dosage, (**c**) influence of initial CR concentration and (**d**) effect of temperature for the adsorption of CR onto SCAC.

As shown in Fig. 5a, a marginal increase in CR removal efficiency was noted between pH 5 and 6, followed by a minor reduction around pH 7. Interestingly, a marginal rise in removal efficiency was observed as the pH continued to increase from 7 to 10. The slight increase in removal efficiency observed between pH 5 and 6 is attributed to the relationship between the pH_PZC_ of SCAC and the pKa of CR^51^. When the pH is below the pH_PZC_, SCAC carries positive charges, while at pH levels above the pH_PZC_, it attains negative charges^52^. On the other hand, CR has a pKa of 4.1, which influences its ionization state in solution. At pH values below its pKa, CR primarily exists in its protonated form, where the amine (–NH_2_) and sulfonic acid (–SO_3_–) groups form NH_3_^+^ and SO_3_H respectively^53^. Within the pH of 5 to 6, the SCAC surface, with a pH_PZC_ of 7.2, is positively charged. In parallel, CR, with its pKa of 4.1, exists predominantly in its deprotonated form. This difference in charge facilitates electrostatic interactions among the charged species, heading to enhanced adsorption performance. However, a slight drop in adsorption efficiency was seen around pH 7, likely due to its proximity to the pH_PZC_ of the SCAC. At this pH, the adsorbent is neutral in charge and thus less likely to attract the negatively charged CR molecules.

Despite the growing negative charge on both the SCAC surface (pH > pH_PZC_) and CR molecules (pH > pKa) at pH 7 to 10, a marginal rise in removal efficiency was achieved. This suggests that non-electrostatic interactions, such as hydrogen bonding among the negatively charged –SO_3_^–^ of CR and the hydroxyl groups of the SCAC surface along with van der Waals forces, become more prominent^53^. Moreover, potential π–π interactions among the aromatic ring of CR and the aromatic structures of SCAC, further enhance adsorption, counteracting the increasing electrostatic repulsion. Considering the meagre variations in removal efficiency across the tested pH range from 5 to 10, the native pH of CR (pH 7.0) was deemed optimal for subsequent adsorption experiments. This decision minimizes the need for extensive pH adjustments, enhancing both the practicality and scalability of the process for real-world applications. The selection of optimum pH resembles findings from previous literature using AC from corn cobs^16^ and watermelon rinds^54^.

#### Influence of SCAC dose

Figure 5b illustrates the correlation between SCAC dose, CR removal efficiency, and adsorption capacity. Specifically, the rise in the dosage from 0.2 to 1.0 g/L caused a dramatic increase in removal efficiency from 25.48 to 89.33%. This positive correlation is corroborated by the increased availability of binding spots, which, in turn, promotes greater interaction and intake of CR molecules, ultimately enhancing their removal^55^. Conversely, the adsorption capacity of SCAC exhibits a consistent decline with increasing dose. In particular, the adsorption capacity was 63.70 mg/g at a dose of 0.2 g/L and decreased to 44.66 mg/g at a dosage of 1 g/L. At higher doses, the abundance of available adsorption sites surpasses the number of CR molecules available for binding. This effect reflects the opposite correlation between dosage and adsorption capacity as given in Eq. (2). Subsequently, an optimum dosage of 0.5 g/L was chosen for further studies. These dosage-dependent findings for CR removal align with previous studies using AC derived from *Sargassum fusiform*^45^ and *Cymbopogon winterianus*^56^.

#### Effects of initial dye concentration and time

The impact of initial dye concentration on the adsorption performance of SCAC was performed by changing the CR concentration between 20 and 60 mg/L while maintaining 0.5 g/L SCAC (Fig. 5c). During the initial 30 min of adsorption, there was a rapid increase in removal efficiency. This initial surge is because of the abundant accessibility of binding sites on SCAC, which readily accommodates CR molecules. As the process continues, equilibrium is gradually reached, marked by a plateau in the removal efficiency curve between 80 and 120 min. This equilibrium state specifies that, the binding sites on SCAC are becoming saturated, limiting further adsorption as the rate of adsorption is counterbalanced by the rate of desorption. Another key observation is the inverse relationship between CR concentration and removal efficiency. With an increase in CR concentration, the removal efficiency dropped from 84.05 to 46.56% over 180 min (Fig. 5c). At lower concentrations, the percentage removal was higher due to the ease with which the CR molecules could access and occupy the abundant active sites^55^. In contrast, the limited number of active sites on SCAC becomes a limiting factor at higher concentrations. Despite the presence of a large number of CR molecules, the active sites are insufficient to accommodate all of them. Consequently, after adsorbing CR up to a certain concentration, the active sites of the SCAC attain saturation. Beyond this saturation point, equilibrium is established, resulting in a decrease in the removal percentage^25^.

#### Influence of temperature

The effect of temperature on the removal efficiency of CR onto SCAC was assessed between 293 and 323 K. As illustrated in Fig. 5d, the removal performance exhibits a clear upward trend with increasing temperature, rising from 39.42% at 293 K to 71.56% at 323 K.

Elevated temperatures enhance adsorption by increasing the kinetic energy of CR molecules, leading to more frequent collisions with the SCAC and providing the thermal energy needed to surpass the activation energy barrier for adsorption^57^. Also, the endothermal nature was evident from the positive correlation between temperature and removal efficiency which aligns with findings from other studies involving AC synthesized from kenaf fiber^55^.

While adsorption is generally considered a less energy-intensive process compared to other separation techniques, conducting it at an elevated temperature of 323 K increases the energy demand, making it less energy-efficient. Furthermore, implementing adsorption at such high temperatures for industrial textile dye wastewater treatment, which involves significantly larger volumes, becomes impractical and potentially cost-prohibitive. Besides, there’s a risk of thermal evaporation of CR solution at high temperatures, leading to an inaccurate estimation of the amount of CR adsorbed by the adsorbent. Therefore, an optimum temperature of 303 K was selected for subsequent adsorption experiments to maintain a synergy between practical feasibility, cost-effectiveness, and environmental sustainability.

### Adsorption kinetics

**Fig. 6:**
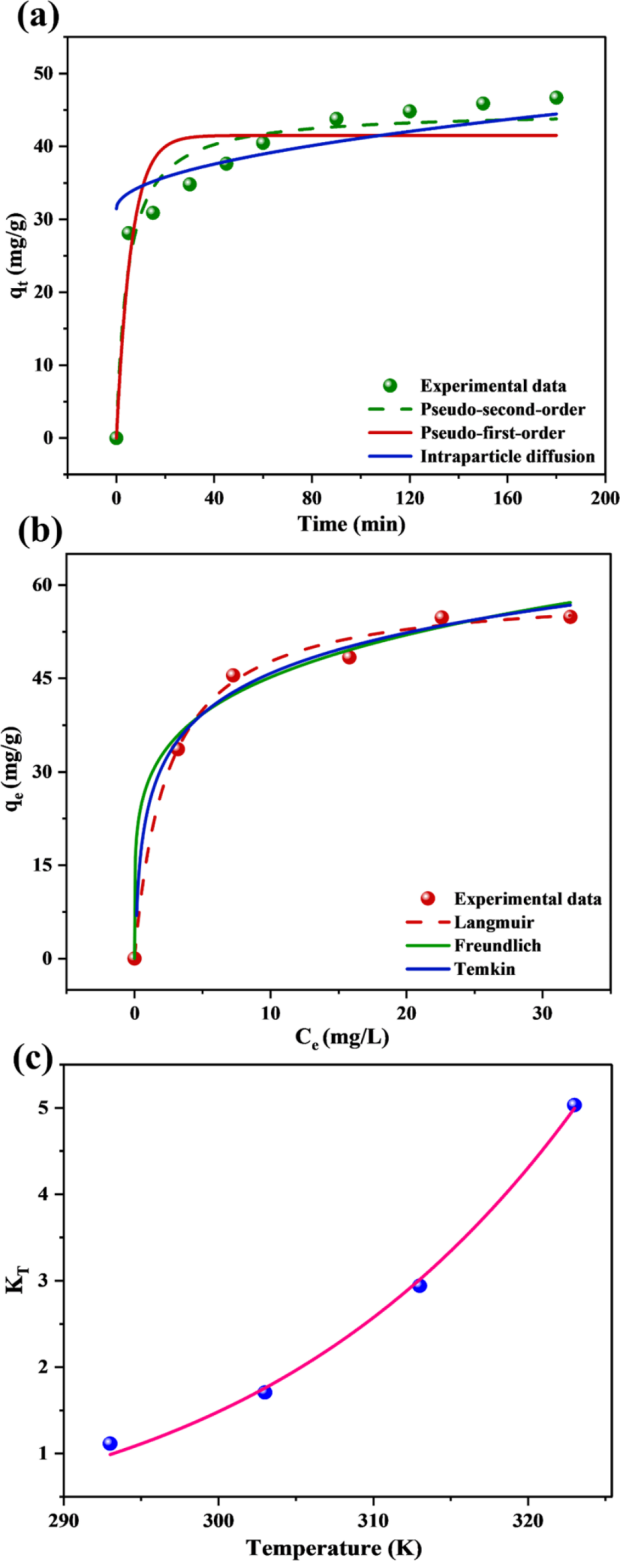
(**a**) Adsorption kinetics, (**b**) adsorption isotherm and (**c**) Van’t Hoff plot for the adsorption of CR onto SCAC.

The adsorption capacity versus time plot reveals a rapid initial uptake of CR within the first 30 min, attributed to the high concentration gradient among the CR solution and the abundant active sites on SCAC (Fig. 6a). This initial rapid uptake is likely driven by physisorption, where CR molecules weakly bind to the SCAC surface through van der Waals forces. As adsorption progresses, the rate decreases, ultimately reaching equilibrium at 180 min. This plateau indicates that the binding sites on SCAC have become saturated^55^.

To gain a deeper understanding of the adsorption mechanism, the dataset was fitted to three well-established kinetic models: PFO, PSO, and IPD as discussed in the earlier sections. The PFO model (Fig. 6a), which assumes that the adsorption rate varies with the accessibility of unoccupied sites, showed a poor fit (R² = 0.8900) (Table 1) to the experimental dataset. Furthermore, the significant deviation between the calculated adsorption capacity (q_e_,_cal_) of 41.50 mg/g and the experimental adsorption capacity (q_e_,_exp_) of 46.67 mg/g implies that the adsorption process includes more complex interactions beyond simple physisorption. This is further supported by the high Chi-square (χ2) value of 24.09, confirming the inadequacy of the PFO model for this system.

**Table 1:** Adsorption kinetics and isotherm parameters for CR adsorption onto SCAC.

	Adsorption kinetics	
Models	Parameters	Values
Pseudo First order Order	$\:{k}_{1}\left({\text{m}\text{i}\text{n}}^{-1}\right)$	0.164
	$\:{\text{q}}_{\text{e}},\:\text{c}\text{a}\text{l}\:$(mg/g)	41.50
	$\:{\text{R}}^{2}$	0.8900
	χ²	24.09
Pseudo Second order Order	$\:{\:\:\:\text{k}}_{2\:\:\:}$(g/mg.min)	0.0048
	$\:{\text{q}}_{\text{e}},\text{c}\text{a}\text{l}$ (mg/g)	44.99
	$\:{\text{R}}^{2}$	0.9544
	χ²	9.99
Intraparticle diffusion	K_diff_ ((mg/g).min ^0.5^)	2.78
	$\:{\text{R}}^{2}$	0.7731
	χ²	49.73
	C (mg/g)	15.19
	Adsorption isotherm	
Langmuir	q_m_ (mg/g)	59.27
	K_L_ (L/mg)	0.411
	$\:{\text{R}}^{2}$	0.9944
	χ²	3.07
Freundlich	K_F_ ((mg/g)/(L/mg)^1/n^	28.34
	1/n	0.202
	$\:{\text{R}}^{2}$	0.9908
	χ²	5.03
Temkin	B_T_ (J/mol)	8.855
	K_t_ (L/mg)	16.95
	$\:{\text{R}}^{2}$	0.9413
	χ²	5.61

In contrast, the PSO model, based on chemisorption, offered a much better fit (R² = 0.9544) (Table 1). Moreover, the calculated adsorption capacity (q_e_,_cal_) of 44.99 mg/g closely aligns with the experimental value (q_e_,_exp_) of 46.67 mg/g, further supporting chemisorption as the governing mechanism^58^. This conclusion is reinforced by the low χ2 value of 9.99, highlighting the minimal difference between calculated and experimental values.

The IPD model was applied to evaluate the diffusion within SCAC pores that contribute to the adsorption process. However, its lower R² (0.7731) (Table 1) and high χ2 value of 49.73 indicate that, although intraparticle diffusion contributes to the adsorption process, it does not serve as the primary rate-limiting step. Besides, the non-linearity in the IPD plot, with an intercept value of C = 15.19 mg/g, indicating the thickness of the boundary layer, further supports the multi-stage nature of the adsorption process. This implies that external mass transfer plays a significant role during the initial phase before intraparticle diffusion becomes the dominant mechanism^59^.

### Adsorption isotherm models

As shown in Fig. 6b, a steep rise in q_e_ was observed at low C_e_ values, indicating a high affinity of CR for SCAC. This initial rapid intake is owing to the abundance of vacant adsorption sites on SCAC. However, as C_e_ increases, q_e_ begins to plateau, suggesting a gradual saturation of the available sites. To further elucidate the mechanism and quantify the interaction between CR and SCAC, the experimental equilibrium data, were fitted to three widely used adsorption isotherm models: Langmuir, Freundlich, and Temkin as discussed earlier.

Langmuir model demonstrated excellent fit to the experimental dataset, evidenced by the high R^2^ value of 0.9944 (Table 1). This strong correlation suggests that the adsorption predominantly follows a monolayer mechanism, characterized by the development of a single layer of CR molecules on the SCAC surface. The Langmuir model assumes that all binding sites possess uniform energy and are equally accessible^52^, which aligns with the observed plateau in q_e_ at higher C_e_ values (Fig. 6b). Furthermore, the Langmuir model yielded a lower χ2 value of 3.07 reinforcing the inference that, it provides a more accurate and reliable fit for the observed adsorption behavior. The K_L_, measured to be 0.411 L/mg suggests a relatively strong attraction between SCAC and CR, indicating a favorable adsorption process. Besides, the separation factor (R_L_), given by, $\:{R}_{L}=\frac{1}{1\:+{\:C}_{i\:}{K}_{L}}$ where, *C*_*i*_ (mg/L) is the initial concentration of CR. It was calculated as 0.108, which falls within 0 < R_L_ < 1 and confirms the favourability of the adsorption process^52^. The maximum adsorption capacity (q_m_), obtained from the Langmuir model was 59.27 mg/g, which surpasses those reported in several other studies using AC for CR removal. For instance, Litefti et al. documented that the adsorption capacity of CR on AC derived from *Pinus pinaster* was 3.92 mg/g^60^. Similarly, Ghaedi et al. reported a maximum adsorption capacity of 10 mg/g for AC derived from *Myrtus communis*^61^. Other AC sources reported in the literature demonstrated varying adsorption capacities for CR such as water hyacinth stem and leaf (14.367 and 13.908 mg/g)^62^, kenaf fiber (14.15 mg/g)^55^, fly ash (22.12 mg/g)^63^ and apricot stone (32.85 mg/g)^64^. Table 2 emphasizes various reported ACs for the removal of CR dye. The data reveals that SCAC stands out with its high surface area, significantly higher pore volume, and maximum adsorption capacity, demonstrating its superior efficacy for CR adsorption compared to several recently reported ACs.

**Table 2:** Analysis of various ACs for CR dye removal.

S. No	Adsorbent	AC preparation conditions	BET surface Area (m^2^/g)	Pore volume (cc/g)	q_m_ (mg/g)	Ref.
1.	*Cymbopogon winterianus* AC	H_3_PO_4_, 300^ο^C, 2 h	-	-	6.25	^56^
2.	Water hyacinth leaf and stem AC’s	500^ο^C, 1 h	3.78	-	13.908	^62^
3.	Prickly Pear Fruit Seeds AC	H_3_PO_4_, 450 ^ο^C, 2 h	42.79	2.34	21.83	^73^
4.	Apricot stone AC	H_3_PO_4_, 450 ^ο^C, 3 h	88.05	0.264	32.85	^64^
6.	*Hevea brasiliensis* seed shells AC	NaCl, 300 °C, 3 h	735	-	50.51	^74^
7.	*Spathodea campanulata* AC	H_3_PO_4_, 400^ο^C, 2 h	986.41	0.8797	59.27	This study

Freundlich model is commonly linked with multilayer adsorption on heterogeneous surfaces^43^. This model deviates from the ideal adsorption scenario by accounting for diverse binding sites with varying adsorption-free energies. Freundlich isotherm also showed a strong correspondence with the dataset, as proven by its high R^2^ value of 0.9908 (Table 1**)**. However, the relatively higher χ2 value of 5.03 obtained from the Freundlich model suggests that the Langmuir model delivers a more accurate representation of the experimental data. Additionally, the Freundlich exponent (*n*) of 4.94 being greater than 1, suggests that the adsorption is favorable^65^. This further supports the conclusion drawn from the Langmuir model, confirming the favorable adsorption. Likewise, a 1/*n* value of 0.202, being relatively low and not approaching 1, suggests a degree of heterogeneity in the adsorption sites^55^.

The Temkin model, which infers a linear reduction in the heat of adsorption with increasing surface coverage caused by adsorbent-adsorbate interactions, offers an additional perspective on the adsorption process. Although its R^2^ value of 0.9413 and χ2 of 5.61 (Table 1) does not indicate a strong correlation as the Langmuir and Freundlich models, it still signifies a reasonable fit to the experimental dataset^52^. Thus, evaluation of the data using Langmuir, Freundlich, and Temkin isotherm model equations suggests a complex adsorption process involving both monolayer and multilayer coverage. This complexity highlights the multifaceted nature of adsorption, often involving a combination of mechanisms and diverse SCAC surface characteristics.

### Thermodynamics studies

The Van’t Hoff plot (Fig. 6c) shows an accurate fit between K_T_ and T with an R² of 0.9972. The calculated negative ΔG° values, specifically − 0.27, − 1.35, − 2.81, and − 4.34 kJ/mol at 293 K, 303 K, 313 K, and 323 K, respectively, indicate that the adsorption becomes progressively more favorable with rising temperature. This trend demonstrates that an increase in temperature enhances the spontaneity of the adsorption process, likely due to the increased kinetic energy of CR molecules and more favorable interactions with the SCAC surface^57^. The ΔH° value of 42.48 kJ/mol reveals that the adsorption process is endothermic, requiring an input of energy. This endothermic nature is consistent with the observed rise in the amount of CR adsorbed at higher temperatures (Fig. 5d). In addition, the measured ΔH^ο^ value exceeds the 40 kJ/mol threshold commonly associated with chemisorption, suggests a chemisorption-driven process as supported by the PSO model. This observed enthalpy change reflects a balance between the initial energy required to overcome solvent interactions and electrostatic repulsion between CR molecules^66^. Furthermore, the ΔS° value of 144.91 J/mol K signifies the improved disorder at the CR/SCAC interfaces. This increase in entropy likely arises from several factors, including the displacement of solvent molecules from the SCAC surface upon CR adsorption^66^. The thermodynamic parameters of this study align with previously published findings on AC derived from *Pterocarpus indicus*^58^ and *Pinus sylvestris* tree bark^29^.

### Desorption and adsorbent regeneration

**Fig. 7:**
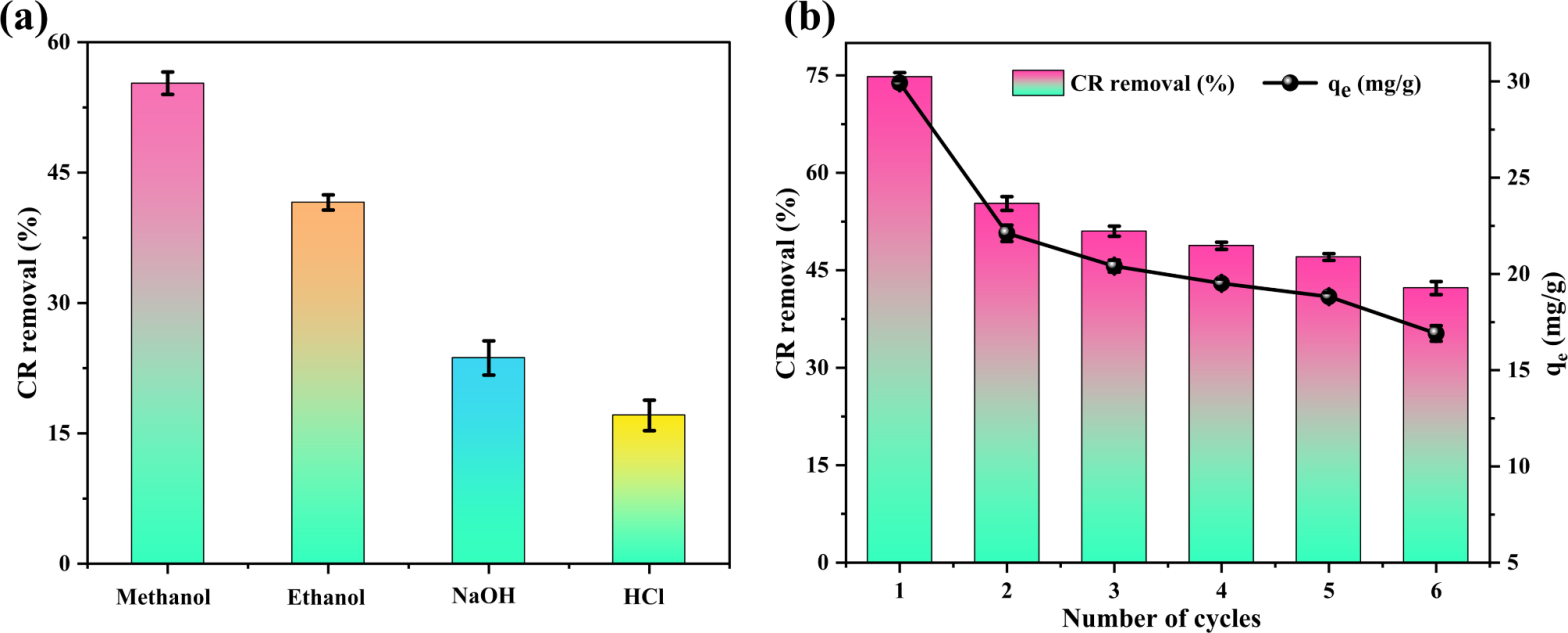
(**a**) Adsorption efficiency of SCAC for CR after using different desorbing agents; (**b**) Adsorption efficiency of SCAC by methanol over six consecutive adsorption-desorption cycles.

The desorption experiments, depicted in Fig. 7a, demonstrate the significant impact of solvent type on CR removal from the spent SCAC. The solvents, in order of decreasing desorption efficiency, were methanol > ethanol > NaOH > HCl with removal efficiencies of 55.28%, 41.56%, 23.66%, and 17.06%, respectively. The superior performance of methanol is due to its lower molecular weight and shorter hydrocarbon chain compared to ethanol. These properties likely facilitate more effective penetration into the pores of the SCAC, disrupting CR-SCAC interactions^67^. Additionally, its higher polarity presumably aids in the disruption of non-covalent interactions like hydrogen bonds or van der Waals force. Conversely, the lower desorption efficiencies observed for NaOH and HCl suggest that the CR-SCAC adsorption is not easily reversed by strong bases or acids. This could be due to the limited pore penetration of larger ionic species, potentially hindered by steric effects or diffusion barriers, or to the formation of insoluble precipitates that retain in the pores upon reaction with CR^68^. Therefore, methanol was selected as the desorbing solvent for regeneration studies.

The regeneration studies with methanol, presented in Fig. 7b revealed a slight decrease in both CR removal efficiency and adsorption capacity with each cycle, yet the SCAC retained a substantial portion of its initial effectiveness. From the first to the second cycle, adsorption performance showed a slight decrease from 74.80% (29.92 mg/g) to 55.28% (22.11 mg/g). However, it remained almost consistent from the second to the sixth cycle, reaching 42.27% (16.90 mg/g) – an overall marginal reduction of only 43.49%. This decline is due to the incomplete desorption of CR molecules, alterations in SCAC surface functional groups, and slight mass loss of SCAC during the regeneration process^69^. Nevertheless, reusability studies demonstrate that SCAC can be effectively regenerated for the removal of CR over multiple cycles, underscoring its potential for sustainable and economical applications in industrial wastewater treatment.

### Evaluating the efficacy of SCAC for removing CR from various water matrices

**Fig. 8:**
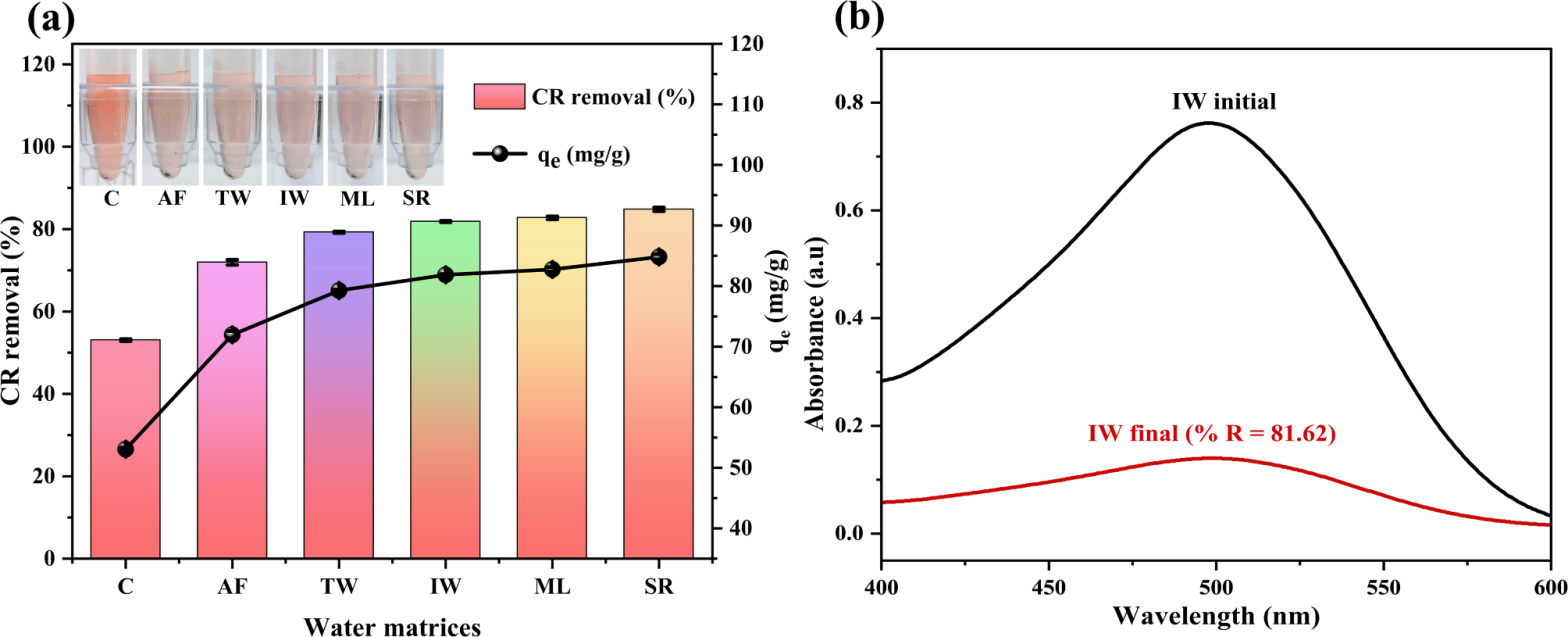
(**a**) CR adsorption performance using SCAC across different water matrices including, Control (C), Arabi falls (AF), Tap water (TW), Industrial groundwater (IW) Manipal lake (ML) and Suvarna river (SR); (**b**) UV-visible spectra of CR removal performance using SCAC in Industrial groundwater (IW).

Figure 8a depicts the CR removal efficiencies and maximum adsorption capacities across different water matrices, highlighting the enhanced performance of SCAC in natural environments compared to the distilled water control (C). Notably, the natural water matrices demonstrated significantly higher adsorption performance in the following order: Arabi falls < Tap water < Industrial ground water < Manipal lake < Suvarna river. Similarly, Fig. 8b illustrates the UV-visible spectra showing the performance of SCAC in industrial groundwater, achieving a removal efficiency of 81.62%, which closely approaches the highest observed efficiency of 84.82% in the Suvarna river sample. This high efficiency underscores the practical applicability of SCAC in complex water sources.

The significantly higher CR removal efficiency observed in various natural water streams is owing to their distinct chemical characteristics. Natural waters typically contain dissolved organic matter, such as humic substances^70^, which can act as bridging ligands between organic dyes like CR and the SCAC surface. Additionally, the presence of various ions in natural water may further influence the surface chemistry of SCAC, favoring adsorption^47^. Since distilled water lacks both organic bridging ligands and ionic diversity present in natural waters, it fails to induce similar surface characteristics, likely contributing to the observed reduction in adsorption efficiency.

### Insights of adsorption mechanism

**Fig. 9:**
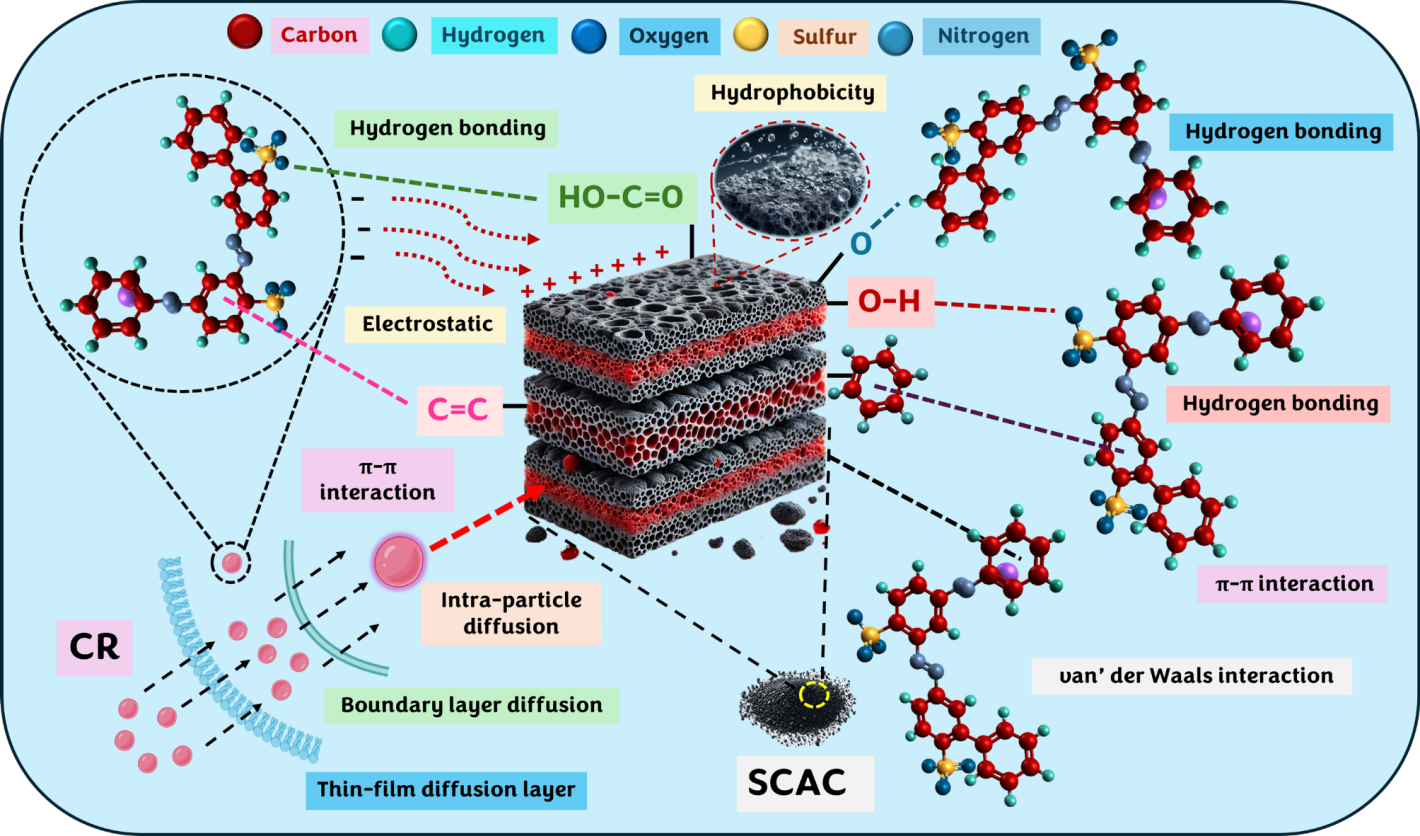
Schematic representation showing the key interactions between SCAC and CR during the adsorption process.

The schematic representation showing the interaction mechanisms between SCAC and CR, is depicted in Fig. 9. The findings of this study suggest that CR adsorption onto SCAC is governed by a multifaceted mechanism, with chemisorption playing a dominant role while physisorption also contributes significantly to the overall process. Initially, the SSA and mesoporous structure provide easy access for CR molecules, enhancing overall adsorption efficiency through physical forces^71^. However, as adsorption progresses, the PSO model demonstrates a superior fit, highlighting the increasing role of chemisorption. Additionally, the presence of functional groups on SCAC, such as ‒OH, C = O, O‒C = O, and aromatic structures, confirmed by XPS and FTIR analyses, facilitate diverse interaction types, including, hydrogen bonding, van der Waals forces and π–π interactions (physisorption), alongside strong ionic/electrostatic (chemisorption). Furthermore, the hydrophobic nature of the aromatic rings and azo groups (–N = N–) in CR molecules contributes to the adsorption through favorable interactions with the non-polar regions of SCAC^72^.

In addition, the superior fit of the Langmuir isotherm confirms monolayer adsorption dominated by chemisorption, while the reasonable fit of the Freundlich isotherm suggests surface heterogeneity and the potential for multilayer adsorption, which is characteristic of physisorption^26^. Additionally, the adsorption process involves a combination of diffusion processes, including bulk diffusion, thin-film diffusion, boundary layer diffusion, and intraparticle diffusion. These processes collectively facilitate the transport of CR molecules from the bulk solution to the internal pores of the SCAC.

At low pH, the positively charged SCAC surface enhances strong electrostatic attraction with the negatively charged sulfonate groups of CR, enhancing adsorption efficiency. At higher pH (7‒10), as the surface charge becomes less positive or even negative, the reduction in strong electrostatic attractions is compensated by non-electrostatic interactions such as van der Waals forces, hydrogen bonding, and π–π interactions. Together, these findings underscore that the surface area, pore structure, pH-dependent charge, and functional groups synergistically contribute to efficient CR removal.

## Conclusion

This study successfully synthesized activated carbon from *Spathodea campanulata* flowers and evaluated its effectiveness for Congo red (CR) removal. FESEM image showed that the initially rough and irregular surface morphology of SCAC transformed to a smoother texture after adsorption, confirming the effective attachment of CR molecules. Further characterization through XPS and FTIR analyses identified surface functional moieties that facilitated adsorption via electrostatic interaction, hydrogen bonding, and π–π interaction. Adsorption studies revealed that CR removal was most effective at pH 7 and a temperature of 303 K, indicating the significance of electrostatic and non-electrostatic interactions driving the adsorption process. The adsorption kinetics followed a pseudo-second-order model, indicating chemisorption as the dominant mechanism, further supported by the ΔH° (42.48 kJ/mol) determined in thermodynamic studies. The Langmuir model provided the best fit, indicating monolayer coverage of CR on a homogenous SCAC surface. Desorption studies demonstrated methanol as an effective desorbing agent, enabling SCAC to retain high adsorption efficiency over multiple cycles, highlighting its reusability. Spiking studies in simulated natural wastewater environments further validated the efficacy of SCAC. Hence, SCAC synthesized from a readily available and sustainable plant biomass, shows great promise as a low-cost and eco-friendly solution to remove CR dye. Its high adsorption capacity and reusability make it a viable option for practical applications. Future research should focus on scaling up SCAC production to industrial levels, expanding its application to contaminants like heavy metals and pharmaceuticals, conducting continuous column studies, evaluating the effects of competing ions on adsorption capacity, cost analysis, and optimizing sustainability through life cycle assessments.

## Data Availability

The authors declare that the data supporting the findings of this study are available within the article.

## Declarations

### Plant guidelines

The authors confirm that the use of plant/plant parts in the present study complies with international, national and/or institutional guidelines.

### Permissions to collect the plants/plant parts

The flowers of the tree were taxonomically identified and authenticated by Ms. Gayathri Pai, Department of Botany, MGM College, Udupi, India. The tree is commonly available and not protected, so no permissions or licenses were required for its collection. Additionally, a voucher specimen has not been deposited in herbarium, considering its established and widespread recognition.

### Source of the plant used in your study

All plant names and the source of them are in the Methods section.
